# Association of parenting with suicidal ideation and attempts in children and youth: protocol for a systematic review and meta-analysis of observational studies

**DOI:** 10.1186/s13643-021-01727-0

**Published:** 2021-08-14

**Authors:** Florence Perquier, Sarah Hetrick, Terri Rodak, Xin Jing, Wei Wang, Katherine T. Cost, Peter Szatmari, Madison Aitken

**Affiliations:** 1 Cundill Centre for Child and Youth Depression, Centre for Addiction and Mental Health, 80 Workman Way, Toronto, ON M6J 1H4 Canada; 2 Department of Psychiatry, University of Toronto, Toronto, ON M5T 1R8 Canada; 3 Department of Psychological Medicine, University of Auckland, Private Bag 92019, Auckland, 1142 New Zealand; 4 Centre for Youth Mental Health, University of Melbourne, Locked Bag 10, Melbourne, VIC 3052 Australia; 5 CAMH Education, Centre for Addiction and Mental Health, 33 Ursula Franklin Street, Toronto, ON M5S 2S1 Canada; 6 Centre for Addiction and Mental Health, 1001 Queen St W, Toronto, ON M6J 1H4 Canada; 7 Department of Psychiatry, Hospital for Sick Children, 555 University Avenue, Burton Wing, Toronto, ON M5G 1X8 Canada

**Keywords:** Parenting, Suicide, Attempted, Suicidal ideation, Child, Adolescent, Systematic review, Meta-analysis, Protocol

## Abstract

**Background:**

Suicide is a leading cause of death in children and youth, with suicidal thoughts and suicide attempts (referred to as non-fatal suicidal behaviors (NFSB)) being among its strongest predictors. Positive parenting (e.g., warmth, responsiveness), negative parenting (e.g., control, hostility), and parent-child relationship quality (e.g., trust, communication) have been reported to be associated with differences in NFSB in this population. To date, no comprehensive systematic review has considered together the wide range of parenting factors studied in relation to NFSB, and no meta-analysis of existing findings has been conducted. The present study will critically appraise and synthesize the existing evidence from observational studies that examine the relationships between parenting factors and (i) suicidal ideation and (ii) suicide attempt in children and youth.

**Methods:**

Studies will be retrieved from APA PsycInfo, MEDLINE, CINAHL, Embase, Scopus, and the Cochrane Library databases. Retrospective, cross-sectional, and longitudinal studies, conducted in clinical and population settings, among youth aged less than 25 years and published as articles and dissertations in English or French will be eligible. Two reviewers will select articles using the Covidence Software after title and abstract screening and full-text assessment, will extract information using double data entry, and will appraise studies’ quality using the Quality Assessment Tool for Observational Cohort and Cross-Sectional Studies. Any disagreements will be discussed with a third reviewer. Publication bias will be evaluated using funnel plots and Egger’s test. In addition to a narrative summary of results, meta-analyses will be conducted using results from at least three studies. Three-level random effect models will allow to derive pooled estimates from dependent effect sizes (from the same sample or study). In case of significant heterogeneity, moderation analyses will be performed considering participants’ characteristics and methodological aspects of studies. The results will be reported according to the PRISMA guidelines, and the certainty of evidence will be assessed using the GRADE approach.

**Discussion:**

In highlighting parenting factors associated with NFSB and in estimating the overall strength of these associations in children and youth, our results will inform further intervention and prevention strategies designed for young people experiencing NFSB and their families.

**Systematic review registration:**

PROSPERO CRD42020165345

**Supplementary Information:**

The online version contains supplementary material available at 10.1186/s13643-021-01727-0.

## Background

Suicide is the second leading cause of death for young people between the ages of 10 and 24 with over 140,000 young people taking their own life each year worldwide [1]. Of concern is the large increase in rates of suicide death and suicide-related behaviors observed among children and youth in the last decade [2]. Notably, the Center for Disease Control and Prevention (CDC) reported that rates of death by suicide increased 56% among US Americans aged 10 to 24 years between 2007 and 2017 [3].

Suicidal ideation, which refers to thinking about, considering, or planning suicide, and suicide attempt, defined as a non-fatal, self-directed behavior, with an intent to die, are among the strongest predictors of future suicide risk [4, 5]. Although suicidal ideation is not considered as a behavior strictly speaking, for easier reading and in line with previous authors, we will henceforth collectively refer to both suicidal ideation and suicide attempt as “non-fatal suicidal behavior” (NFSB) [6, 7]. Rates of onset of NFSB increase sharply from late childhood to peak during late adolescence and early adulthood [8, 9], and intervention for young people experiencing these phenomena is therefore widely recognized as being an important part of suicide prevention strategies.

Existing interventions and prevention strategies for children and youth show promising results in reducing the frequency of NFSB and self-harm (the latter referring to any self-injurious behavior with or without the underlying intent to die, including suicide attempts) [10, 11]. However, their effectiveness may be limited in part because their designs remain largely based on available evidence in adults. To ensure strategies are as effective as possible, it is essential to adapt interventions to the needs of children and youth by tackling specific risk and protective factors for NFSB in this population. In particular, theoretical assumptions, along with a growing body of literature, suggest that parenting is a crucial factor to be considered [12–14].

From a theoretical standpoint, the stress-diathesis model of suicide provides a useful framework to understand the putative role of parenting in the development of NFSB, in combination with other biological, cognitive, psychological, social, and environmental factors [15]. According to this model, suicidal behavior results from the interaction of an individual vulnerability (e.g., genetic or psychological), or *diathesis*, along with exposure to proximal stressor(s), such as psychiatric disorders and stressful life events.

Parenting is involved in both *diathesis* and stress components of this model and may influence the risk of NFSB in a negative or in a positive way. Early childrearing environment exerts a formative influence on children’s vulnerability to stress (*diathesis*). For example, early supportive parenting and mutual parent-child interactions promote the formation of secure attachment and contribute to the regulation of negative emotions in children, which in turn have been reported as protective factors against NFSB [16, 17]. Parenting factors may also contribute to stress, especially when the child is growing up. Some negative parenting factors, such as harsh punishment or abuse, act as proximal stressors, which can precipitate the development of NFSB in combination with a preexisting *diathesis* [18]. Conversely, positive parenting might moderate or counteract the effect of other stress factors on NFSB in vulnerable people. For instance, in a cohort study of 550 US adolescent females, high levels of parental support were found to be protective against suicidal ideation following exposure to a stressful life event [19].

This theoretical account also suggests that the risk of NFSB in children and youth might be prevented or reduced by adapting parenting information and support to caregivers’ and their children’s needs. Such support should aim at reducing parenting factors highly associated with the risk of NFSB, while enhancing those recognized as having a beneficial effect. Most interventions for NFSB already involve a parent component, but to date, there is still insufficient evidence with regard to the effects of specific parenting programs or which parenting components to target as a priority.

Various parenting factors have been examined in relation to NFSB. These factors are commonly categorized as (i) parenting practices, which refer to specific behaviors that parents use in raising a child, and (ii) aspects of the parent-child relationship, which capture the broader emotional climate (e.g., closeness, communication, or attachment) created by the reciprocal interactions existing between the child and the parent [20, 21].

Parenting practices can further be grouped into the two broad categories of positive and negative parenting, based on the consistency of the associations of parenting practices with respectively positive and negative outcomes in children and adolescents [22]. Positive parenting commonly refers to positive control and warmth (e.g., monitoring, supervision, consistent discipline, involvement, support), whereas negative parenting is characterized by high levels of negative control and hostility (e.g., overprotection, rejection, harsh parenting, coercion). It appears useful to mention here that these categories do not assume any a priori association between the pertaining parenting factors and NFSB.

**Fig. 1:**
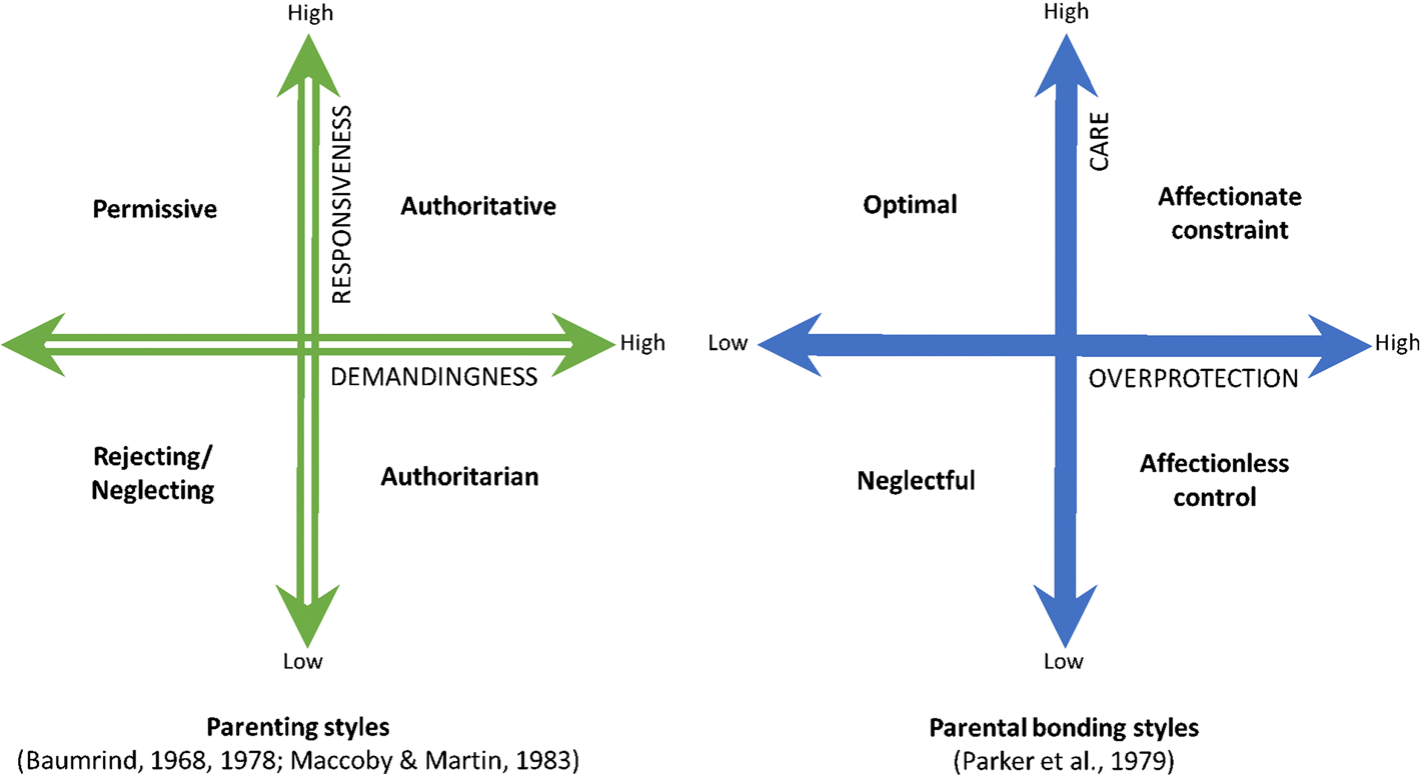
Parenting styles and parental bonding styles

Some specific parenting practices have also been combined to derive *parenting styles* [23–25] and parental bonding styles [26] (Fig. 1). In accordance with the definition of positive and negative parenting mentioned above, the authoritative parenting style and optimal bonding style, characterized by high levels of warmth, care, positive control, and low levels of negative control, are considered positive parenting [27], while other parenting styles and parenting bonding styles are commonly considered as negative parenting.

To date, four literature reviews, but no meta-analysis, have synthesized evidence regarding the relationship between parenting and NFSB in adolescence, focusing on parenting styles and parental bonding styles [28–31]. They have demonstrated good evidence of a protective role of parental warmth and care, and a detrimental association of parental neglect, authoritarian parenting, and affectionless-control parental bonding style with NFSB [28–31]. The specific associations of permissive parenting and of affectionate-constraint parental bonding style with NFSB remain, however, unclear [29].

Unfortunately, findings from these literature reviews are limited in reflecting the extended evidence existing on the relationship between parenting and NFSB for two main reasons.

First, previous reviews considered only a small number of parenting factors related to parenting styles and parental bonding styles and did not synthesize the findings focusing on some specific positive parenting practices, such as parental support or monitoring of children’s activities [32, 33], or on some negative parenting practices, such as physical and emotional abuse [34] or role reversal [35]. Moreover, other aspects of the parent-child relationship such as parent-child conflicts, attachment problems, emotional unavailability of parents, poor communication, and low connectedness have been reported to be associated with suicidal ideation [36–38] and suicide attempts [36, 39, 40], but to our knowledge, evidence regarding these factors has never been systematically reviewed.

Second, conclusions made by the existing reviews rely only on studies conducted in adolescents, which may not be generalizable to children or to emerging adults. Indeed, the associations of some parenting factors with child and youth outcomes have been shown to vary according to different developmental stages. Regarding the depression, for example, autonomy granting and monitoring emerged as a relevant factor to consider in adolescence but not as much in childhood [41, 42].

We intend to address these gaps in conducting a systematic review examining the relationships of parenting with NFSB in children and youth considering an extensive range of parenting factors together. In addition, we will conduct the first meta-analysis on the topic, in order to estimate the overall strength of the associations between parenting factors and NFSB, to better characterize the heterogeneity in results obtained in existing studies and the role of potential moderator factors.

### Objectives

Our study will synthesize observational evidence regarding the relationships between parenting and two distinct outcomes—suicidal ideation and suicide attempt—in children and youth. We will answer the following research question: “In children and youth, is parenting associated with suicidal ideation and suicide attempt based on observational quantitative evidence?”

## Methods/design

We will undertake a systematic review of existing observational evidence and will perform meta-analyses where sufficient data are available. The study has been pre-registered with AsPredicted (No. 39505) and registered to the International Prospective Register of Systematic Reviews (PROSPERO CRD42020165345). This protocol follows the Preferred Reporting Items for Systematic Review and Meta-Analyses for Protocols (PRISMA-P) 2015 guidelines (see Additional file 1) [43].

### Eligibility criteria

The inclusion/exclusion criteria have been defined according to the Population of interest, Exposure, Comparator, and Outcome (PECO) statements as described below.

Our population of interest will be children and youth aged less than 25 years old, with no geographical limitation. We will include studies carried out in clinical and population settings among individuals aged less than 25 years or whose mean age is under 25. We will exclude studies that do not specify participants’ mean age or age range. No lower age limit will be set in order to inform the scientific community about the youngest ages considered in existing studies and about potential research gaps and limits in very young children.

The term “parent” will refer to the biological or adoptive parent(s), guardian(s), or caregiver(s).

In accordance with the rationale described earlier and with previous meta-analyses examining the relationship of parenting with child outcomes, parenting factors will be classified according to the three broad categories of positive parenting, negative parenting, and parent-child relationship [44–46].

Studies will be eligible for inclusion if they assess parenting before the age of 18 or at a mean age lower than 18. We set this age limit knowing that, in most countries and states, reaching 18 years corresponds to legal emancipation of children and is marked by more autonomy, life decisions, and often changes in living arrangement that have a main impact on how parents and children perceive the role of parenting and the parent-child relationship [47].

We will focus on the following two outcomes as defined by the CDC: (i) suicidal ideation, which refers to thinking about, considering, or planning suicide, and (ii) suicide attempt, which refers to a non-fatal, self-directed, potentially injurious behavior with intent to die as a result of the behavior [5].

We will not consider data related to non-suicidal self-injury (NSSI) and non-suicidal self-harm in the present study. Although they are highly comorbid with suicidal behaviors in children and adolescents, these are phenomenologically different [48] and could be influenced by distinct protective and risk factors [49].

Some studies have examined the relationship of parenting with self-injury and self-harm, which refer to any self-injurious behavior, including suicidal (suicide attempt) but also non-suicidal self-injurious behavior (NSSI and non-suicidal self-harm).

In order to identify all relevant data pertaining to suicide attempts, our search strategy is meant to capture these studies by including relevant keywords such as “self-injury,” “self-harm,” or “self-mutilation.” However, we will make a distinction between suicidal and non-suicidal self-injurious behavior based on the presence of an intent to die as a result of the behavior, in accordance with the CDC’s definition of suicide attempt given above and with the standardized nomenclature established based on the Columbia Classification Algorithm of Suicide Assessment [9, 50]. During the article selection process, reviewers will carefully assess the definition of each outcome considered (including in sub-analyses) and will only include studies reporting data on suicide attempts, defined as committed with at least some intent to die as a result of the act. Studies that examine self-harm as a single entity without differentiating suicidal from non-suicidal self-injurious behavior, as well as those in which the intent to die is not ascertained, will be excluded, because we assume that they do include non-suicidal behaviors.

Only observational studies with retrospective, cross-sectional, or longitudinal designs will be eligible for inclusion. We chose to exclude case-control studies for two main reasons. First, they are more prone to selection bias when control subjects are not selected from the same population as the cases [51]. Second, in suicide research, the risk of recall bias might be higher in case-control than in cross-sectional studies. Indeed, in case-control studies, NFSB participants are typically recruited and information collected in the days following suicidal behavior, when parents and their offspring often try to make sense of it [52]. Therefore, they might recall parenting factors in more detail and overreport them compared to controls, which might artificially strengthen the observed associations with NFSB.

To examine the bidirectional association of parenting and NFSB, we will include longitudinal studies that examine either the effect of parenting on subsequent suicidal behavior or the effect of suicidal behavior on subsequent parenting factors. The findings of case reports, case series, therapy/treatment-based intervention studies, discussion articles, exclusively qualitative studies, reviews, or meta-analyses will be excluded. However, the reference lists of literature reviews and meta-analyses will be reviewed to capture possible additional relevant citations.

Studies published (or “in-process”) in a peer-reviewed journal as well as dissertations will be included. The inclusion of dissertations will allow us to consider results published outside of traditional commercial publishing and thus reduce the risk of publication bias [53]. However, we will not include conference posters and presentations for two reasons. First, they may not contain adequate information about the study design, methods, biases, and results, limiting critical appraisal of corresponding studies. Second, the association of parenting and NFSB has already been examined in a large number of studies published as articles and dissertations, and in that case, the inclusion of conference abstracts in meta-analyses has been shown to result in only small differences in the effect estimates [54].

Our research team includes members who are proficient in English and in French, making us able to review research works published in these two languages.

### Search strategy

A primary search strategy was developed in APA PsycInfo by a health sciences librarian (TR), and after review and validation by co-authors, the final search strategy was run in APA PsycInfo on November 6, 2019 (Additional file 2). On the same day, it was translated and applied in MEDLINE, CINAHL, Embase, Scopus, and the Cochrane Library databases and was also run in MEDLINE Epub Ahead of Print and In-Process & Other Non-Indexed Citations, to capture the most recent literature. Database-specific subject headings and keywords in natural language were used to capture “parenting dimensions” and “suicidality” concepts, and combined using Boolean logic and operators including proximity searching. These results were then limited to articles where “child” and “adolescent” terms and their synonyms appear in selected fields, and to observational study types. No year limits or language limits were applied.

### Data screening

Two principal independent reviewers (FP and XJ) will follow a two-step selection process using the Covidence® software, according to the eligibility criteria described previously. The first decision will be made based on the titles and abstracts. Then, the selected articles will be considered for full-text assessment to determine if they definitely qualify for inclusion. Any disagreement will be discussed by the two reviewers, and any remaining discrepancies will be resolved by a third reviewer (MA).

### Data extraction

Data will be extracted separately by the two principal reviewers using a standardized data extraction form and a coding process implemented in the Research Electronic Data Capture System (REDCap®). The following information will be systematically extracted from the included studies:

- General study characteristics: first author, year of publication, journal, and type of publication (peer-reviewed article or dissertation).
- Study setting: country where the study was performed and setting in which it took place (mental health care setting, other clinical care settings, or population-based).
- Study design: type of study (e.g., cross-sectional, longitudinal) and time period for data collection.
- Sample characteristics: sample size, age of participants (range and/or mean ± standard deviation) or corresponding school grades, gender distribution, and main ethnicity (defined as the ethnicity shared by more than 60% of participants, otherwise ethnicity will be defined as “balanced”),
- Measurement of parenting: type of parenting, measurement time frame, type of informant (child, parent, other), relationship of caregiver with the child (biological parents only or not), and method for assessment (questionnaire, interview, or observation).
- Assessment of NFSB: type of outcome (suicidal ideation or attempts), assessment time frame, informant (child, parent, other), and method for assessment (questionnaire, interview, observation).
- Effect estimates: non-adjusted and/or adjusted effect estimates (along with their standard deviation or 95% confidence intervals) relating to the association of each parenting factor with one or both of our outcomes will be extracted and converted to odds ratios (OR) for dichotomous outcomes and standardized mean differences (Cohen’s d) for continuous outcomes, using conventional conversions.

Any disagreements between the two extraction processes will be resolved by consensus discussion with the third reviewer. In case of unclear or incomplete data, original authors will be contacted.

### Risk of bias

The two principal reviewers will independently assess the methodological quality of studies using the Quality Assessment Tool for Observational Cohort and Cross-Sectional Studies developed by the US National Heart, Lung and Blood Institute (NHLBI) [55]. This validated tool includes 14 items for evaluating potential bias induced by study methods or implementation, including patient selection, attrition, confounding, sample size justification, and arguments for causation. Reviewers will select “yes,” “no,” or “cannot determine” in response to each item. Some questions of the tool have been slightly adapted to better capture the strengths and weaknesses of existing studies in the scope of our topic. Reviewers will also rate the overall study quality as “good,” “fair,” or “poor” based on their rating for each item and their own critical appraisal of the risk of bias, as recommended by the guidance document developed by the NHLBI methodology team. If reviewers rate the overall quality of the study as poor, they will state the reasons for the decision. In case of disagreements, consensus will be sought through discussion between raters and, if necessary, with the third reviewer.

### Data synthesis

Evidence regarding the association of parenting factors with each of the two outcomes (suicidal ideation and suicide attempt) will be reported according to the Preferred Reporting Items for Systematic Review and Meta-Analysis (PRISMA) criteria [43] and satisfy the Meta-analysis Of Observational Studies in Epidemiology (MOOSE) Checklist for Meta-analyses of Observational Studies [56].

Meta-analyses will be performed using a random effect model when a minimum of three studies with usable data are available. We will calculate the effect sizes as odds ratios (OR) or standardized mean difference (Cohen’s d), with standard errors, and convert information reported in a different metric using conventional conversions.

A narrative summary of the evidence will be provided by outcome, including results from studies that would not be possible to consider in meta-analysis. The results will be presented using forest plots and in a “summary of findings table.” The Grading of Recommendations Assessment, Development, and Evaluation (GRADE) approach will be used to rate the certainty of the evidence [57]. Risk of bias (assessed as previously described), inconsistency of results, indirectness of evidence, imprecision of effect estimates, and risk of publication bias will be considered as reasons to rate down the quality of evidence, whereas a large magnitude of effect and the presence of a dose-response gradient will be considered to rate it up.

Studies often report on multiple effect sizes obtained from the same sample or in the same epidemiological study, for instance, when examining the associations of different parenting factors with NFSB or when considering the measures reported by different informants (mother/father or youth). In that case, we can assume that the corresponding effect sizes are dependent [58], and it is inappropriate to perform a standard meta-analysis because the assumption of conditional independence of effect sizes is violated [59]. A strategy could be to consider only one effect size per study and to perform separate meta-analyses for each type of exposure [60]; however, this implies that some associations are more valid or of greater priority and results in a loss of information. Moreover, it becomes impossible to examine the moderation effects between several exposures of interest (in our case, between parenting factors). The use of three-level models has thus been recommended to model dependent effect sizes without losing available information, especially in studies examining the role of different parenting factors that could influence each other [46]. Our three-level meta-analyses will allow us to consider (1) the effect size level, (2) the sample level, and (3) the study level.

The study design is known to influence the strength of the observed associations, especially since parenting is likely to influence NFSB and NFSB can also have an impact on parenting [61]. As results from cross-sectional studies do not allow these bidirectional associations to be disentangled, we will investigate their results separately from those obtained in longitudinal studies, while distinguishing longitudinal studies that examine the effects of parenting on NFSB from those studying the consequences of NFSB on subsequent parenting.

Heterogeneity will be assessed by visual inspection of forest plots, Cochrane’s Q, and Higgins’ test (I^2^). The I^2^ values, corresponding to the observed heterogeneity that would not be expected by chance, will be classified as low (< 30%), moderate (30–50%), and severe (> 50%) [62].

In case of significant heterogeneity, we will conduct moderator analyses considering the participants’ characteristics and methodological aspects of studies. Associations between parenting and NFSB were previously reported to differ according to age [63], child assigned sex [64, 65], and ethnicity [66]. Moreover, parenting takes place in a broader cultural and socio-political context, which differs widely according to participants’ countries of residence. Countries, as well as their income level defined by the World Bank as low- and middle-income countries (LMIC) and high-income countries (HIC), have been shown to influence the risk of suicide behaviors in children and youth [67, 68]. Among methodological aspects, the methods used for the assessment of parenting and NFSB (e.g., using questionnaire or observation data) and whether the informant is the child or the parent could also explain some differences observed in previous findings [28, 29]. We will thus consider participants’ age, sex, and ethnicity; countries and their income level; methods for assessment; and informants as potential moderators in our meta-analysis.

Publication bias will be evaluated through visual inspection of funnel plots and by using Egger’s test. The “trim and fill” method will be applied to correct for publication bias [69].

### Sub-group and sensitivity analyses

If possible, sub-group analyses will be conducted according to different study settings (mental health care setting, other clinical setting, or population-based).

To examine whether the inclusion of studies with the highest risk of bias might affect our results, and in accordance with the Cochrane Handbook, sensitivity analyses will be performed by restricting the primary analysis to studies at low risk of bias, after exclusion of those whose quality was rated as “poor” on the Quality Assessment Tool for Observational Cohort and Cross-Sectional Studies. When possible, sensitivity analysis comparing the results between meta-analyses of adjusted and unadjusted data will be conducted to inform about the presence of confounding.

We will identify the effect size outliers, defined as effect sizes falling more than 2.2 standard deviations away from the pooled result, as well as small sample size outliers (n < 100) [70]. These outliers will be considered in a leave-one-out sensitivity analysis, which consists of performing separate meta-analyses on each subset of the studies obtained by iteratively leaving out one outlier [71].

Analyses will be performed using comprehensive meta-analysis and R.

## Discussion

Despite the diversity in parenting approaches, a growing body of literature suggests that some parenting practices and aspects of the parent-child relationship influence the risk of NFSB in children and youth [28–31]. Interventions and policies that promote parenting factors that are the most beneficial while reducing those having the most deleterious effects may thus contribute to lowering the risk of NFSB in children and youth.

A deep understanding of the specific parenting factors associated with NFSB is thus required, including providing an estimation of the strength of the corresponding associations and examining whether and how they vary in different populations or according to studies’ methodologies. Our systematic review will synthesize the findings of observational studies considering the association of various parenting factors (including positive or negative parenting, as well as aspects of the parent-child relationship) with suicidal ideation and suicide attempts in children and youth. Besides, we will conduct the first meta-analysis on the relationship of parenting with NFSB, enabling us to present the associations as pooled estimates and to examine the heterogeneity between studies.

To date, the conceptual framing of parenting as well as the available evidence regarding the relationship between parenting and NFSB mostly come from high-income countries (HIC). It is possible that the results obtained in these countries are not similar to those obtained in low- and middle-income countries (LMIC), due to the differences in socio-economic contexts [72] and cultural differences in the perception of NFSB or in how children respond to the parenting factors and differential access to mental health services [73]. Fortunately, a non-negligible number of studies have been conducted in children and youth from LMIC, using data from original studies [74, 75] and from the Global School-based Student Health Survey (GSHS) [76–78]. Their inclusion in our study will allow us to highlight the possible discrepancies between LMIC and HIC in the association of parenting and NFSB.

NSSI and non-suicidal self-harm may represent points along the continuum of self-harm and are associated with the risk of future NFSB [79]. While we recognize the importance of better addressing the relationship between parenting and these behaviors in order to prevent self-harm in children and youth, we excluded NSSI and non-suicidal self-harm from the scope of the present review because they are distinct from suicidal attempt in terms of clinical presentation, motivations, and etiology [48, 49]. Moreover, given suicidal ideation and suicide attempts are strongly predictive of suicide death, we considered that focusing on these two outcomes would provide the most effective opportunities to prevent suicide in children and youth. The exclusion of some studies that have used broad definitions like “self-harm” or “self-injury” without distinguishing suicidal from non-suicidal self-injurious behaviors could lead to a loss of information but will ensure that our outcomes are accurately defined. We also note here that our results would not be generalizable to the relationship between parenting and self-harm or self-injury in children and youth.

Another limitation could reside in the small number of available studies considered in some of our meta-analyses. Running a meta-analysis with at least three studies will allow us to present, for the first time, pooled estimates for various parenting factors in relation to NFSB. It corresponds to the median number of studies usually included in meta-analyses from the Cochrane Database of Systematic Reviews [80]. Herbison et al. have examined the change in validity of pooled estimates with the accumulation of evidence over time [81]. With three studies, the 95% confidence interval included the final estimate in 72% of meta-analyses and the inclusion of more studies did not dramatically change the estimates. In our case, the majority of studies eligible for inclusion rely on large population samples, which should ensure sufficient statistical power. However, having a small number of studies could affect the assessment of heterogeneity and of publication bias in our study. In consequence and in accordance with the GRADE approach [57], we will systematically report the number of studies and participants included in each meta-analysis, as well as the quality of evidence available, and discuss any limitation due to each of these factors in the final manuscript.

Our findings could be of great interest for health professionals working with children and youth with NFSB and their families. They should inform and enhance the intervention efforts, by highlighting parenting factors that might be important targets for intervention or that could be useful to understand as mechanisms of actions of interventions in this high-risk population. The impact of various therapeutic interventions on suicidal and non-suicidal self-harm, including interventions focusing on young people, family-centered interventions, and interventions targeting wider social networks of the young people has been synthesized by Ougrin et al. [11]. Those with the largest effect sizes were found to be dialectical behavior therapy (DBT), cognitive-behavioral therapy (CBT), and mentalization-based therapy (MBT), especially when a family component is included. Understanding which aspects of parenting are worth targeting in priority when addressing NFSB will help to refine intervention strategies while ensuring they are cost-effective and efficient.

From a preventive point of view, our results could also emphasize the need of supporting parenting at a population level. They may have implications for policymakers and public health specialists regarding the development of universal prevention programs able to promote beneficial parenting skills (through early parenting training or public health messaging, for example). Furthermore, they might help to tailor selective prevention programs according to the specific needs of population sub-groups and to specific countries’ contexts.

Our study will also pinpoint research gaps and future research priorities regarding the association between parenting and suicidal ideation and suicide attempt in children and youth. Our findings could represent a new frame for reference for future research on this specific topic but also inform research on familial transmission of NFSB [82]. According to the results of a previous meta-analysis, children whose parents have a history of suicide attempt are at increased risk (OR = 2) of attempting suicide [83]. Recent results from O’Reilly et al. suggested that, in addition to genetic factors and comorbid parental behavioral health problems, approximately 15% of the intergenerational association of suicidal behavior is due to environmental mediation [84]. Parenting factors have been identified as key mediators in the familial transmission of depression and antisocial behaviors and could play a significant role in the transmission of suicidal behavior as well [85]. Although our study will not focus on the parent-child transmission of suicidal behavior per se, we will be able to identify the parenting factors that would be interesting to consider as potential mediators in future studies.

In summarizing and communicating the evidence on the topic, our study will contribute to the translation of evidence-based knowledge required to encourage the development of promising studies in this critical research area, guide clinical practice, and support the development of policies in the treatment and prevention of suicidal behaviors in children and youth.

## Supplementary Information

**Additional file 1:.** PRISMA-P 2015 Checklist.

**Additional file 2:.** Search Strategy in APA PsycInfo database – searched on November 6th, 2019.

## Data Availability

Not applicable

## Abbreviations

CAMH: Center for Addiction and Mental Health
CDC: Center for Disease Control and Prevention
GRADE: Grading of Recommendations Assessment, Development, and Evaluation
NFSB: Non-fatal suicidal behavior
NSSI: Non-suicidal self-injury
NHLBI: National Heart, Lung and Blood Institute
MOOSE: Meta-analysis Of Observational Studies in Epidemiology
OR: Odds ratio
PECO: Population of interest, Exposure, Comparator, Outcome
PRISMA: Preferred Reporting Items for Systematic Review and Meta-Analysis
PRISMA-P: Preferred Reporting Items for Systematic Review and Meta-Analysis Protocols
PROSPERO: International Prospective Register of Systematic Reviews
REDCap: Research Electronic Data Capture System
SMD: Standardized mean difference
US: United States
