# Supplementary material for: Association of parenting with suicidal ideation and attempts in children and youth: protocol for a systematic review and meta-analysis of observational studies
Source: Syst Rev. 2021 Aug 14;10:232. doi: 10.1186/s13643-021-01727-0 (PMC8364684; doi:10.1186/s13643-021-01727-0)
Supplement: Supplementary file 2 — Additional file 2:. Search Strategy in APA PsycInfo database – searched on November 6th, 2019. [file 13643_2021_1727_MOESM2_ESM.docx]

**Additional file 2: Search Strategy in APA PsycInfo database – searched on November 6th, 2019.**

1 exp parenting style/

2 exp childrearing practices/

3 exp Parent Child Relations/

4 exp parenting/

5 ((Parent* or mother* or father* or maternal* or paternal*) adj3 (Style* or dimension* or behavio?r* or relationship*)).ti,ab,id.

6 ((Parent* or mother* or father* or maternal* or paternal*) adj3 (bond* or warmth or warm or authorit* or neglect* or permissive* or restrict* or supervis* or monitor* or connect* or accept* or support* or discipline* or reject* or hostil* or domina* or submissi* or detach* or involve* or respons* or control* or overcontrol* or protect* or overprotect* or demanding*)).ti,ab,id.

7 or/1-6 [parenting dimensions]

8 exp suicide/

9 suicidal ideation/

10 (suicid* adj3 (attempt* or commit* or complet* or die* or dead or ideation* or thought* or plan* or consider* or contemplat* or behavio?r*)).ti,ab,id.

11 exp self-injurious behavior/

12 (selfharm* or self-harm or selfinjur* or self-injur* or selfinflict* or self-inflict* or self-mutilat* or selfmutilat* or selfpoison* or self-poison*).ti,ab,id.

13 ((fatal* or lethal* or intentional* or deliberate*) adj2 (dose or doses or dosing or overdos* or self-administ* or selfadminist*)).ti,ab,id.

14 or/8-13 [suicidality]

15 (Child* or boy or boys or girl* or kid or kids or offspring or minor or minors or adolescen* or juvenil* or youth* or young adult* or teen* or emerging adult* or transition* age* or college age* or university age* or underage* or under-age* or pubescen* or prepubescen* or pediatric* or paediatric* or student*).ti,ab,id,hw,po.

16 7 and 14 and 15

17 limit 16 to ("0400 empirical study" or "0430 followup study" or "0450 longitudinal study" or "0451 prospective study" or "0453 retrospective study" or "0600 field study" or 1800 quantitative study or 2200 twin study)

18 (quantitative or observational or retrospective or cohort* or cross-sectional or case control* or longitudinal or quantitative).ti,ab,id,hw.

19 ((empirical or prospective or field or twin or followup or follow-up) adj1 (study or studies)).ti,ab,id,hw.

20 16 and (18 or 19)

21 17 or 20 [final set]

22 (nonsuicid* not suicid*).ti,ab,id,hw.

23 21 not 22 (1022)

***************************
